# Decay of Airborne Bacteria from Cattle Farm Under A-Band Ultraviolet Radiation

**DOI:** 10.3390/ani14243649

**Published:** 2024-12-18

**Authors:** Luyu Ding, Qing Zhang, Ligen Yu, Ruixiang Jiang, Chunxia Yao, Chaoyuan Wang, Qifeng Li

**Affiliations:** 1 Information Technology Research Center, Beijing Academy of Agriculture and Forestry Sciences, Beijing 100097, China; dingly@nercita.org.cn (L.D.); yulg@nercita.org.cn (L.Y.); jiangrx@nercita.org.cn (R.J.); yaocx@nercita.org.cn (C.Y.); 2 National Engineering Research Center for Information Technology in Agriculture (NERCITA), Beijing 100097, China; 3 National Innovation Center of Digital Technology in Animal Husbandry, Beijing 100097, China; 4 Department of Agricultural Structure and Bioenvironmental Engineering, College of Water Resources and Civil Engineering, China Agricultural University, Beijing 100083, China; zhqinger@foxmail.com (Q.Z.); gotowchy@cau.edu.cn (C.W.)

**Keywords:** airborne bacteria, A-band ultraviolet, decay rate, dynamic inactive rate, laboratory simulation

## Abstract

Inspired by the effects of solar or UV radiation on the decay of airborne bacteria during their transport, this study investigated the effect of UVA on the decay of airborne bacteria from cattle houses and analyzed the potential use of UVA to reduce indoor airborne bacteria under laboratory conditions. Airborne bacteria from the cattle source were generated and released into a small-scale test chamber (1.5 m^3^) with different strategies according to the different objectives in decay tests and simulated sterilization tests. Increasing with the UVA radiation gradients (0, 500, 1000, 1500 μW cm^−2^), the average decay rate of total curable airborne bacteria ranged from 2.7% to 61.6% in decay tests. Under the combination of different UVA radiation intensities (2000 μW cm^−2^ in maximum) and radiation durations (60 min in maximum), simulated sterilization tests were conducted to examine the potential use of UVA radiation for air sterilization in animal houses. With the dynamic inactive rate (DIR) ranging from 17.2% to 62.4%, we proved that UVA may be an alternative way to reduce the indoor airborne bacteria in cattle houses if applied properly. Similar effects would be achieved using either a high radiation intensity with a short radiation duration or a low radiation intensity with a long radiation duration.

## Simple Summary

A-band ultraviolet (UVA) in solar radiation plays an important role in the natural decay of airborne bacteria. In this study, laboratory simulation experiments were conducted to investigate the impact of varied UVA intensities on the decay of airborne bacteria and to analyze the potential use of UVA to reduce indoor airborne bacteria in cattle houses. Decay and inactive rates of airborne bacteria from cattle sources increased with the UVA radiation intensities when the radiation was over 1500 μW cm^−2^, and it was affected by the initial concentration and radiation durations. Results of this study provide the foundation for further applying UVA as an alternative way to reduce indoor airborne bacteria in animal houses.

## 1. Introduction

The increase in livestock facilities has led to an increase in airborne bacteria emissions from the stalls into the environment and aroused a wide concern in the public [1,2,3,4]. The presence of animals in the stall discharges a large number of bacteria from the skin scales, breath, urine, feces, and secretions to their surroundings [5]. After being discharged, bacteria can persist in the air as individual cells or attach to solid or liquid particles to be bioaerosols, becoming airborne in the environment [2,6]. Once aerosolized, airborne bacteria transport with the airflow and can remain in the atmosphere for a significant period of time, threatening the health of animals or humans by inhaling into the respiration system [3,7,8]. During transport, the quantity and activity of airborne bacteria are reduced due to sedimentation and biological decay, which are greatly affected by meteorological factors [9,10].

Previous studies showed that a wide range of environmental factors, including solar radiation or UV intensity, precipitation events, and humidity, would affect the patterns of airborne bacteria during their transportation [2,11]. Under natural conditions, sunlight or solar radiation has a certain bactericidal and disinfection effect, which mainly depends on the invisible light waves of ultraviolet (UV). UV is separated into four bands, which are UVA (400–320 nm), UVB (320–380 nm), UVC (280–200 nm), and vacuum ultraviolet (VUV, 200–80 nm) [12]. Due to the absorption and blocks of the ozone layer in the atmosphere, over 90% of ultraviolet rays that reach the near ground are UVA, and the rest are UVB. Although there is limited information on UVA in air sterilization, it has been proven that UVA can be a novel method applied to water sterilization for purposes such as the disinfection of the nutrient solution residual liquid of a hydroponic system in greenhouses or drinking water disinfection [13,14,15]. This suggests that UVA would affect the decay of airborne bacteria and can be a potential method for indoor sterilization of livestock houses to reduce the airborne bacteria being transported to outdoor environments as it is less biologically destructive than UVC and also has the effect of promoting the formation of vitamins.

Previous studies on the UV inactivation of airborne bacteria mainly focused on UVC. For example, Christopher et al. [16] assessed the ability of UVC with a predominant wavelength of 254 nm to disinfect individual bioaerosols of airborne bacteria in the bioaerosol chamber. Results show a 99% inactive rate for *Escherichia coli*, *Micrococcus luteus*, *Pseudomonas fluorescens*, and *Staphylococcus aureus*, and an inactive rate of over 75% for *Bacillus subtilis*. Kim and Kang [17] investigated the inactivation rate of virus, bacterial, and fungal aerosols in a chamber-type air disinfection system by using a UVC light-emitting diode array. They showed a varied inactivation rate constant that ranged from 0.28 to 2.64 cm^2^ mJ^−1^ for different microbial species and different UVC doses in the range of 0–25 mJ cm^−2^. In addition, the bactericidal effect of the specific band of UV is related to radiation intensity and radiation duration. Taking UVC as an example, the time required to kill viruses and bacteria, mold spores, and algae bacteria is 0.1–1.0 s, 1.0–8.0 s, and 5.0–40.0 s, respectively, at the UVC radiation intensity of 30 μW cm^−2^ [18]. As there is a source specificity of airborne bacteria in different origins, it is necessary to assess the potential bactericidal effect and dose of UVA, both radiation intensity and radiation time, to the application scenarios before use.

Quite a bit of research had been conducted on the concentration and emission of airborne bacteria or bioaerosols from livestock houses. Although there is a relatively high indoor concentration of airborne bacteria due to the high density of animals in the closed pig and poultry houses, cattle farms may have a higher outdoor concentration of airborne bacteria at the farm level. There is a large variation in measured indoor total bacteria, mostly in the range of 10^3^ to 10^7^ in pig and poultry houses [3,19], whereas results showed that the farm-level and surrounding concentration of total bacteria were less than 2000 CFU m^−3^ [20]. This may be because of the requirement and practice of centrally treating the exhausted air to reduce environmental pollution. Differing from the centralized mechanical ventilation in pig and poultry houses, cattle are normally raised in a naturally ventilated, free-ranging system with an unorganized airflow pattern. This makes it hardly possible to do exhaust gas treatment in the cattle house, and airborne bacteria are easily transported to the outdoor environment. Compared to that in pig and poultry farms, a relatively higher farm-level and surrounding concentration of total bacteria (3000–4000 CFU m^−3^) was observed in cattle farms [21], although there was a relatively lower indoor total bacteria observed (mostly in the range from 10^3^ to 10^4^) in cattle houses [22]. Moreover, field measurement showed that the concentration of airborne bacteria in cattle houses would intermittently exceed the standard requirement when the ventilation area of the cattle barn is reduced in winter [23]. Hence, it would be important to find a way applicable to cattle houses to reduce the emissions of airborne bacteria in the cattle house.

Inspired by the effects of solar or UV radiation on the decay of airborne bacteria during their transport, this study assessed the decay rate of airborne bacteria under varied UVA radiation and investigated the possibility of inactivating airborne bacteria from cattle farms with different doses of UVA radiation in laboratory conditions. The results of this study would provide a foundation for further application of UVA for air sterilization in livestock houses and enrich the knowledge and understanding for evaluating the effects of UV radiation on the decay of airborne bacteria after emitting from animal houses.

## 2. Materials and Methods

In this study, an experimental setup was established to simulate the decay process of airborne bacteria emitted from cattle barns. The change in airborne bacteria concentration over time in the experimental setup was monitored to investigate the bactericidal effects of UVA radiation and analyze the possibility of its application on air disinfection in cattle houses.

### 2.1. Experimental Setup and Airborne Bacteria Generation

**Figure 1:**
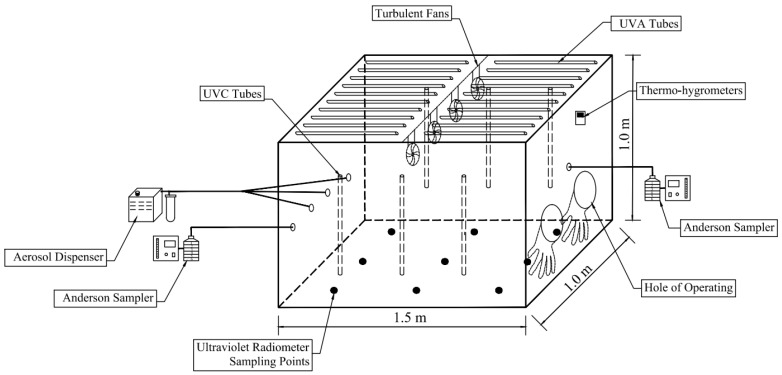
The schematic diagram of the experimental setup.

Figure 1 shows the schematic diagram of the experimental setup. It mainly includes an acrylic test chamber equipped with UVA and UVC tubes, an aerosol dispenser (HY-220, Beijing Zhongbang Industrial Technology Co., Ltd., Beijing, China), and the six-stage Anderson samplers (TISCH, TEI Corp., Hamilton, OH, USA). The literature showed that aerosol test chambers may vary in size from regularly 1–2 cubic meters to rooms approximately 30–50 cubic meters [24]. Chambers with a capacity of around 1 m^3^ were normally used in existing research. For example, Paez-Rubio and Peccia estimated the solar and nonsolar inactivation rates of airborne bacteria with a 1 m^3^ chamber using a sampling flow rate of 12 L min^−1^ [25]. Hasegawa et al. [26] studied the bacterial culturability during bioaerosol challenge using a test chamber of 1 m^3^ at a low sampling flow rate of 20–28.5 L min^−1^ and using a test chamber of 3 m^3^ at a high sampling flow rate of 100 L min^−1^. The test chamber in this study is 1.5 m long, 1.0 m wide, and 1.0 m high, with a capacity of 1.5 m^3^. There are eight air nozzles evenly distributed on both sides of the chamber for aerosol generation, gas sampling, and air exchange. Rubber gloves are installed with holes on one side of the chamber to facilitate the experiment operation in the test chamber when it is closed. Totally twenty UVA tubes (340 nm, 600 mm long, 40 W), which can run independently, were installed on the top of the test chamber. Turbulent fans are installed in the chamber for gas distribution, and six UVC tubes are installed on two side walls of the chamber for inner sterilization before and after each experimental test. Air temperature and relative humidity in the chamber were logged using a recorder (179A-TH, Apresys Inc., Los Angeles, CA, USA) every 5 min during the experiment.

According to field measurements and the literature reports, the daily maximum ultraviolet radiation intensity in the Beijing–Tianjin–Hebei region can reach 2000 μW cm^−2^, and most of them are A-band ultraviolet (UVA) [27]. Thus, UVA gradients were set at the expected radiation intensity of 2000 μW cm^−2^ at maximum. Different UVA intensities were simulated by controlling the number of UVA tubes turned on in the test chamber to reach the expected ultraviolet. Actual UVA intensities were measured by an ultraviolet radiation meter (wavelength range: 320–400 nm and λ_P_ is 365 nm; SDR2040, Shenzhen Speedre Technology Co., Ltd., Shenzhen, China). Before conducting experiments, UVA intensities were measured at nine evenly distributed points 50 cm vertically from the top of the chamber when different number of UVA tubes were operated in the test chamber. To seek the proper operated numbers of UVA tubes at the expected UVA radiation level, UVA intensities were measured with the operated tubes in a gradient of one, five, ten, fifteen, and twenty at the very beginning. Then, the operated number of tubes was fine-tuned to measure UVA intensities. The operated tubes were evenly distributed during each time. For example, denoting the UVA tubes on the left column from 1 to 10 and the UVB tubes on the left column from 11 to 20 in Figure 1, tube 5 was turned on when one tube was operated, and tubes 2, 4, 5, 7, 9, 12, 14, 16, 17, and 19 were turned on when ten tubes were operated. There were four repeated measurements for each gradient, and the UVA intensities were represented using the average of four repeated measurements in nine points.

Airborne bacteria were generated and sprayed into the test chamber using the aerosol dispenser with the medium of the extraction solution from cow manure and deionized water. The concentration of airborne bacteria in the cattle house ranged from 2.43 × 10^3^ to 2.01 × 10^5^ CFU m^−3^ according to the field measurements [23]. A preliminary experiment was conducted to explore the proper ratio between manure and deionized water to obtain a similar concentration range of airborne bacteria in the test chamber. Based on the preliminary experiment, the extracting solution used for airborne bacteria generation was prepared by dissolving 2 g of fresh manure in 60 mL of deionized water and filtering with gauze to make the extraction solution. The working flow rate of the aerosol dispenser varied for airborne bacteria generation in two parts of the experiments and is described in the following sections.

### 2.2. Decay Test of Airborne Bacteria Under Different UVA Radiations

In the decay test, the concentration of airborne bacteria in the chamber was measured at different times to calculate the decay rate after the bioaerosol was released. Airborne bacteria decayed in both physical and biological means. Physical decay is the physical elimination of a particle from the air by means of a series of processes such as gravitational sedimentation, impaction, and electrostatic precipitation, and biological decay is the loss of biological activity of an airborne microorganism [28]. Normally, the measured decay rate is the result of both physical and biological decay, and this study mainly focused on the total decay rate.

Airborne bacteria were generated at a flow rate of 4 L min^−1^ and sprayed into the test chamber for 20 min in each test. After stopping the release of airborne bacteria, the test chamber was kept closed for 60 min, and air samples were collected every 20 min by the six-stage Anderson samplers with disposable nutrient agar plates (every 1000 mL of nutrient agar contains: peptone 10.0 g, beef extract 3.0–5.0 g, sodium chloride 5.0 g, and agar powder 12.0–14.0 g). Two replicate air samples were taken at the sampling flow rate of 28.3 L min^−1^ for 2 min each time from two sides of the test chamber. Samples were incubated for 24 h at the temperature of 37 °C, and colonies in sampling plates were counted with a counter (Icount-30F, Xunshu Technology Co., Hangzhou, China). The correction of colony numbers and concentration calculation of airborne bacteria were performed using the methods as described in Ding et al. [23]. The effect of UVA on the microbial decay of airborne bacteria was investigated by comparing the decay rates under different UVA radiations of 0, 500, 1000, 1000, and 1500 μW cm^−2^. Decay rate (*N_t_*) was calculated using the concentrations of airborne bacteria in the test chamber at different times through Equation (1).

(1) $$N_{t}=\frac{\left(C_{0}-C_{t}\right)}{C_{0}}\times 100\%$$

where *C*_0_ is the initial concentration of airborne bacteria in the test chamber, CFU m^−3^; *C_t_* is the concentration of airborne bacteria in the test chamber at time *t* after initial, CFU m^−3^; *N_t_* is decay rate, which represents the ratio of the number of microbes dying per unit time in a population to the total number of microbes in that population, %.

### 2.3. Simulated Sterilization Test

Animals and their excreta are the main sources of airborne bacteria emission in animal houses. This is a dynamic equilibrium of a process that, between microbe decay and continuing to produce, emits airborne bacteria in the emission scenarios of a closed or semi-closed cattle barn. To simulate the emission scenarios and investigate the reduction effect of airborne bacteria in cattle houses, airborne bacteria were generated and sprayed into the test chamber at a flow rate of 4 L min^−1^ for 20 min and then switched to a minor airflow rate (1.5 L min^−1^) for constant generation to maintain a relatively stable concentration during the following experimental period.

Comparative tests were conducted when there was a treatment of UVA irradiation with different intensities (2, 500, 1000, 1500, 2000 μW cm^−2^) and without UVA irradiation (control) in the test chamber. The test chamber was irradiated with UVC tubes for 20 min to kill the background microorganisms in the chamber before artificial generation of airborne bacteria, and samples were also collected to obtain the background concentration of airborne bacteria (denoted as sample T_0_, it should be zero theoretically) in the test chamber before the experiment. The secondary sampling was conducted when the airflow rate was switched to 1.5 L min^−1^ to obtain the initial concentration (denoted as sample T_1_), and different numbers of UVA tubes were turned on at this time for the treatment tests. Then, samples were collected every 15 min for a total of 60 min, and samples were denoted as samples T_2_, T_3_, T_4_, T_5_, and T_6_, respectively. Samples were collected and incubated using the same method as described in Section 2.2. Each treatment had three replicates, and control tests were conducted in each UVA radiation gradient. As there is a natural decay of airborne bacteria, the dynamic inactivation rate *(DIR*) of airborne bacteria was introduced and calculated using Equations (3) and (4) to quantify the bactericidal effect of UVA. Moreover, UVA radiation dose (*RD*, J m^−2^) was calculated using Equation (2) to further evaluate the bactericidal effect of UVA considering both radiation intensity and radiation time.

(2) RD=RI×t

(3) $$\eta_{1}=\frac{{c_{T_{1}-}c}_{T_{n}}}{c_{T_{1}}}\times 100\%$$

(4) $$DIR=\frac{c_{T_{1}}^{\prime }\left(1-\eta_{1}\right)-c_{T_{n}}^{\prime }}{c_{T_{1}}^{\prime }\left(1-\eta_{1}\right)}\times 100\%$$

where η_1_ is the natural decay rate of airborne bacteria, which is calculated from the control test, %;$\;c_{T_{1}}$ and $c_{T_{n}}$ are the concentration of airborne bacteria initial and at a certain moment in the control test, respectively, CFU m^−3^; *DIR* is the dynamic inactivation rate of airborne bacteria for UVA, %; and $c_{T_{1}}^{\prime }$ and $c_{T_{n}}^{\prime }$ are the concentration of airborne bacteria initial and at a certain moment in the treatment tests, respectively, CFU m^−3^; *RI* is the radiation intensity of UVA, W m^−2^; *t* is the radiation duration, s.

### 2.4. Data Processing and Analysis

Due to a relatively large magnitude and variation in measured total airborne bacteria, concentration of the measured airborne bacteria was expressed as mean ± standard error. Meanwhile, the remaining factors were expressed as mean ± standard deviation in this study. These were air temperature, relative humidity, UVA intensity, decay rate, etc. The significance test of difference was performed to analyze the decay rates of airborne bacteria as well as the DIR of UVA under different treatments. Regression analysis of influencing factors was performed to investigate the impact of environmental factors on the microbial decay of airborne bacteria. Linear regression between DIR and radiation dose was performed to obtain the bactericidal curve in the cattle using UVA irradiation to guide practical application. Moreover, a machine learning model based on the Random Forest algorithm (RF) was built to further analyze the sensitivity of decay rate to the influence factors by coding in Python (code is available in the Appendix A). In the RF model, the influence factors were set as input variables and the decay rate was set as output. The 10-fold cross-validation was adopted to fully utilize the dataset and avoid overfitting or underfitting. All inputting variables are analyzed to obtain the extent to which each independent variable explains the dependent variable.

## 3. Results and Discussion

### 3.1. Measured Environment Condition in the Experimental Setup

To determine the proper operation tube number for the required target intensity gradients of UVA radiation, this paper examined the relationship between the number of ultraviolet tubes and the radiation intensity. Different UVA intensities of 2000, 1500, 1000, 500, and 2 µW cm^−2^ were simulated and achieved through a varied number of UVA tubes in the experimental setup, which were 20, 15, 10, 6, and 1, respectively. Table 1 shows the measured UVA intensity and the actual number of tubes operated at the target intensity of UVA radiation. The UVA intensity gradient in the following text is expressed by the expected.

During this study, the background air temperature and humidity were about 23.7 ± 3.3 °C and 32.3 ± 5.7%, respectively. Due to the heating effect of UVA tubes after operation, there will be a slight temperature rise of about 2 °C with the increase in time in the experimental setup. Even if the same media preparation and aerosol generation methods are used, the initial concentration of airborne bacteria in the experimental setup will still vary. For example, the measured initial concentration of airborne bacteria ranged from 6000 to 9000 CFU m^−3^ when the bioaerosol was generated for 20 min at an airflow rate of 4 L min^−1^ using the liquid medium of cattle manure extraction (manure/deionized water = 1.0 g/60 mL). Thus, the initial concentration of generated airborne bacteria, air temperature, and relative humidity were given, and their effects on the decay of airborne bacteria were analyzed in the following section.

### 3.2. Decay of Airborne Bacteria

After stopping the release of airborne bacteria with the aerosol dispenser, the concentration of airborne bacteria in the experiment setup decreased with time. The decay rates of airborne bacteria were calculated from measured concentrations at different times after airborne bacteria release. The obtained decay rates of airborne bacteria ranged from 2.7% to 61.6%, respectively, in this study. Table 2 shows the average decay rates of airborne bacteria under varied UVA radiation. Statistical analysis showed that the decay rate without UVA radiation was significantly lower than that with UVA radiation of 1500 µW cm^−2^ (*p* < 0.05). No significant differences were observed for the decay rates of airborne bacteria when the target UVA intensity was 500 and 1000 µW cm^−2^ (*p* > 0.05). While decay rate was higher under the target UVA radiation intensity of 1500 µW cm^−2^ (*p* < 0.05).

**Figure 2:**
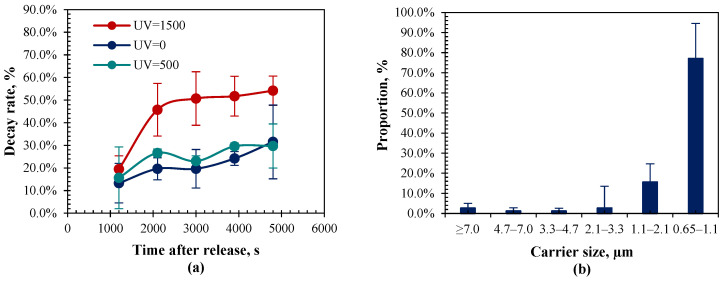
Measured decay rates and carrier size of the airborne bacteria aerosolized into the chamber. (**a**) Decay rates under varied UVA radiation intensities; (**b**) Size distribution of the generated airborne bacteria (1500 µW cm^−2^).

Figure 2a shows the curve of decay rate along with the time after the release of airborne bacteria. The curve of decay rates is very close to each other between the target UVA radiation of 0, 500, and 1000 µW cm^−2^. For a better visual display, Figure 2a only shows the curve of decay rates at the target UVA radiation of 0, 500, and 1500 µW cm^−2^. At the same condition, the decay rates of airborne bacteria tended to increase rapidly with time at the very beginning and then remain relatively stable. Taking the experimental condition at target UVA radiation of 1500 µW cm^−2^ as an example, its average decay rate rose from 19.5% to 45.7% within the time from 20 min to 35 min after the release of bioaerosol, whereas its decay rate stabilized at about 50% within the time from 50 min to 80 min after the release of bioaerosol. Figure 2b gives the size distribution of the airborne bacteria aerosolized into the chamber at the target UVA radiation intensity of 1500 µW cm^−2^. The generated airborne bacteria were mainly distributed in the fine particles whose diameter was less than 2.1 µm. The measured proportion of carriers was 77.1 ± 17.5%, 15.6 ± 9.1%, 2.7 ± 10.9%, 1.3 ± 1.3%, 1.3 ± 1.5%, and 2.7 ± 2.4% in the size range of 0.65–1.1 µm, 1.1–2.1 µm, 2.1–3.3 µm, 3.3–4.7 µm, 4.7–7.0 µm, and ≥7.0 µm, respectively.

Furthermore, the initial concentration affects the decay rate of airborne bacteria in the experimental setup. A higher decay rate was observed at a higher initial concentration of airborne bacteria. Still taking the experimental condition at target UVA radiation of 1500 µW cm^−2^ as an example, the average decay rate was 52.2 ± 15.5% over 80 min after the release of bioaerosol when the initial concentration of airborne bacteria was 8715 CFU m^−3^ in the chamber, while those were 45.5 ± 15.2% and 35.4 ± 13.2% when the initial concentration of airborne bacteria was 6684 and 5358 CFU m^−3^ in the chamber. The previous literature showed that environmental factors like T_a_, RH, and solar radiation would affect the decay rate [25,29,30]. Paez-Rubio and Peccia [25] estimated the solar and nonsolar inactivation rates of airborne bacteria to produce decay curves under moderate (50–60%) and high (85–95%) levels of RH. Their results showed that RH was an important environmental factor for controlling the inactivation of airborne bacteria. Comparing with the high RH level of 85–95%, the greatest inactivation rates were observed at a moderate RH level of 50–60%.

**Figure 3:**
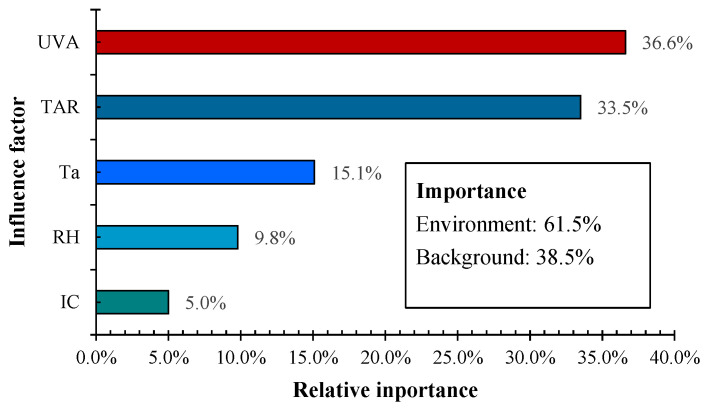
Relative influence of the impact factor on the decay rate of airborne bacteria.

As mentioned above, the initial concentration (IC), UVA intensity, T_a_, and RH in the experimental setup, as well as time after release (TAR), would affect the decay rate of airborne bacteria. To further analyze the impacts of these factors, a statistical model based on a random forest algorithm was developed to relate the bioaerosol concentrations to these impact factors using the measured data in previous sections. The root mean square error (RMSE) was 6.8%, and the R^2^ was 0.81, which indicates the good reliability of the developed model. Results showed that UVA intensity was the most important factor affecting the decay rate in this study. The relative influence of the surveyed factors was ranked in the order of UVA > TAR > T_a_ > RH > IC. These impact factors are further divided into two categories, i.e., environmental factors (T_a_, RH, UVA) and background factors (IC, TAR). Using the feature importance analyzed through RF algorithms, the importance of influence factors to the decay was plotted in Figure 3. As shown in Figure 3, environmental factors had a greater impact on the decay of airborne bacteria, while the influence of background conditions cannot be ignored, especially the TAR.

### 3.3. Effectiveness of UVA on Airborne Bacteria Sterilization

**Figure 4:**
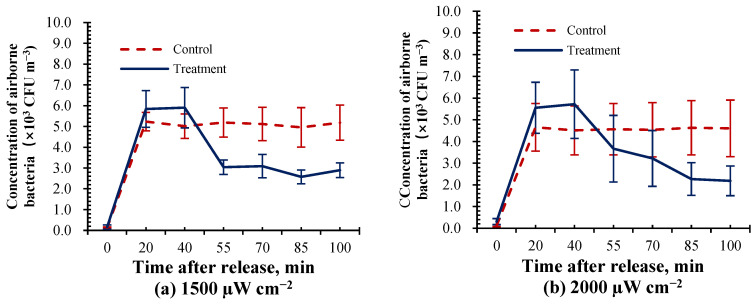
Concentration of airborne bacteria between control and UVA treatment in test chamber. (**a**) UVA radiation intensity at 1500 μW cm^−2^; (**b**) UVA radiation intensity at 2000 μW cm^−2^.

According to the above results, this study analyzed the DIR of airborne bacteria under different radiation intensities to investigate the effectiveness of UVA on airborne bacteria sterilization. Figure 4 shows an example of the concentration of airborne bacteria between control and UVA treatment in the experiment setup at the radiation intensity of 1500 and 2000 µW cm^−2^. As shown in Figure 4, the concentration of airborne bacteria under control in the test chamber remained in a stable state when the flow rate changed to 1.5 L min^−1^ and 20 min after airborne bacteria were artificially generated. Compared with the control group, the concentration of airborne bacteria reduced under the treatment of UVA at different radiation intensities. This suggests that there is a bactericidal effect of UVA in the test chamber, and the specific bactericidal efficiency was further calculated.

**Figure 5:**
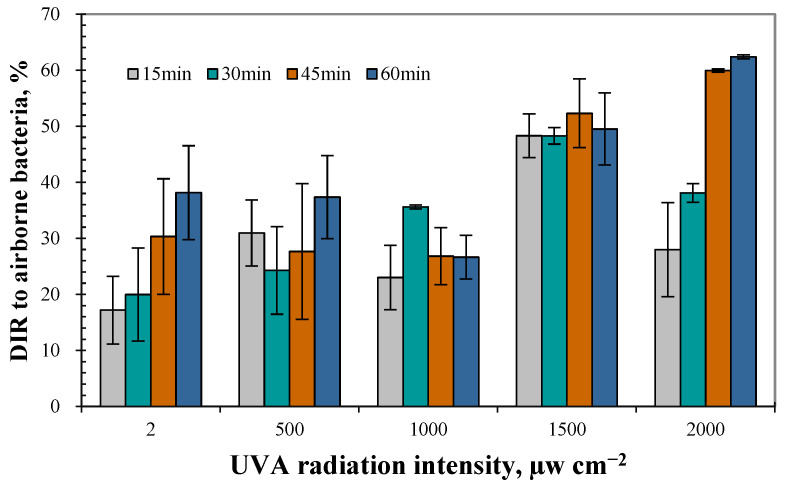
Dynamic inactivation rate (DIR) of airborne bacteria at different radiation intensity and radiation duration of UVA.

Ranging from 17.2% to 62.4%, Figure 5 shows the DIR of UVA to airborne bacteria at different radiation intensities and radiation durations. Much higher DIRs of airborne bacteria was observed when the radiation intensity was over 1500 μW cm^−2^ (*p* < 0.05), while there was no significant difference between the DIR when the radiation intensity was 2, 500, and 1000 μW cm^−2^ (*p* > 0.05). The DIR of airborne bacteria ranged from 28.0% to 62.4% at the radiation intensity of 2000 μW cm^−2^ and positively relates to the increased radiation durations, which averaged 47.1%. Under the radiation intensity of 2 μW cm^−2^, it also showed a positive correlation between DIRs and radiation duration, and the average DIR was 26.4% during the whole experimental duration of 60 min. However, DIR showed little change with radiation duration under the radiation intensity of 500, 1000, and 1500 μW cm^−2^, whose average DIR was 28.0%, 31.0%, and 49.6%, respectively.

Comparing with UVC, UVA is less biologically destructive, and its bactericidal ability is not as strong as UVC. For example, the research from Lin et al. [31] showed a bactericidal rate of 64–82% for UVC under the radiation intensity of 640 µW cm^−2^ in the ventilation ducts with different air speeds (2–3 m s^−1^) and lining materials (polished aluminum plate, aluminum-plastic plate, galvanized iron sheet, and black cloth). This is much higher than the DIR observed in this study and consistent with the results of previous studies [32]. However, there is a dual effect of air sterilization and promotion of vitamin D synthesis for UVA [23]. UVA tubes may be a clean and safe way to reduce indoor airborne bacteria in the presence of animals and improve animal health, especially for newborn animals like calves.

As mentioned before, radiation duration would affect the bactericidal effect of UVA. Radiation dose was introduced to present the compound effect of radiation duration and radiation intensity. The relationship between radiation dose and dynamic inactive rate of UVA was further analyzed. DIR increased with RD and was linearly correlated with RD (*p* < 0.01). This is consistent with the results from Wang et al., who investigated the inactivation efficiency of *Escherichia coli* at an exposure UV dose of 370 J m^3^ under UVA (365 nm), UVC (254 nm), and UVD (185 nm) sources [32].

**Figure 6:**
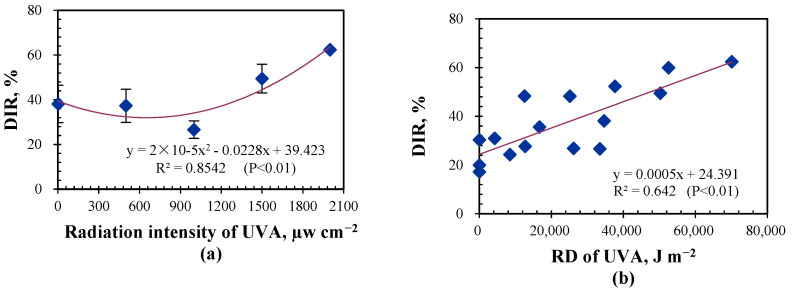
Fitting curve (**a**) between DIR and radiation intensities of UVA at an irradiation for 60 min, and (**b**) between DIR and the radiation dose of UVA.

Figure 6b illustrates the linear regression curve of DIR to RD and of DIR to radiation intensity. Taking the RD of 36,000 J m^−2^ as an example, this could be a combination of the radiation intensity of 2000 µW cm^−2^ with the radiation duration of 15 min or the combination of the radiation intensity of 500 µW cm^−2^ with the radiation duration of 60 min. The measured DIR was 28.0% at the radiation intensity of 2000 µW cm^−2^ with the radiation duration of 15 min and was 37.3% at the radiation intensity of 500 µW cm^−2^ with the radiation duration of 60 min. Based on the regression curve, DIR would be 33.4% at the RD of 18,000 J m^−2^, which is close to the mean (32.6%) of measured DIR at the radiation intensity of 2000 µW cm^−2^ with the radiation duration of 15 min and the radiation intensity of 500 µW cm^−2^ with the radiation duration of 60 min. This suggests that similar DIR would be achieved using either a high radiation intensity with a short radiation duration or a low radiation intensity with a long radiation duration.

Based on the fitting curve, the radiation dose should be over 169,000 J m^−2^ if a DIR of 60% is to be achieved when applying to cattle houses. This can be a radiation intensity of 50 µW cm^−2^ to be irradiated for an hour or a radiation intensity of 565 µW cm^−2^ to be irradiated for 5 min, depending on the animal’s tolerance and costs of this approach, etc. One thing is worth noting, it can be inferred from the results in Section 3.2 that the background concentration of airborne would affect the DIR, and a higher DIR may be achieved at a higher background concentration of airborne bacteria. The concentrations of airborne bacteria in cattle houses, especially in winter, can easily exceed the background concentrations used in this study. Optimistically, the DIR may be even higher in practical application in a cattle house.

## 4. Conclusions

Exposure to airborne bacteria emitted from intensive livestock farming has drawn wide concern among the public. The risk of exposure relates to the decay of airborne bacteria during their transportation. This study investigated the effect of UVA on the decay of airborne bacteria and analyzed the potential use of UVA to reduce indoor airborne bacteria in cattle houses under laboratory conditions. Airborne bacteria were generated and released into the test chambers with different strategies according to the different objectives in decay tests and simulated sterilization tests. UVA can increase the decay rate of airborne bacteria from cattle sources when it reaches a certain radiation intensity, and this would be affected by background conditions (TAR, IC, etc.). The obtained decay rates of airborne bacteria ranged from 2.7% to 61.6% under the varied UVA radiations of 1500 μW cm^−2^ in the maximum. The results of this study proved that UVA had a bactericidal effect, and its dynamic inactive rates (DIR) ranged from 17.2% to 62.4% under the varied UVA radiation intensities in the maximum radiation durations of 60 min. Even the UVA intensity of 2 μW cm^−2^ can achieve a good reduction in airborne bacteria from cattle sources under a sufficient radiation duration. The UVA intensity of 1500 μW cm^−2^ can be adopted when it is used for a short duration of UVA radiation.

## Figures and Tables

**Table 1:** Measured UVA intensity under the target simulation gradients.

Target UVA Intensity (μW cm^−2^)	Number of Operated UVA Tubes	Measured UVA Intensity (μW cm^−2^)
2000	20	1947.8 ± 10.0
1500	15	1396.2 ± 0.4
1000	10	930.3 ± 0.2
500	6	476.5 ± 0.1
2	1	2.1 ± 0.0
0	0	/

**Table 2:** Average decay rate under varied UVA radiation intensities (the same letter in the upper right corner indicates a significant difference for decay rate).

Measured UVA Intensity (μW cm^−2^)	T_a_ (°C)	RH (%)	IC ^1^ (CFU m^−3^)	Decay Rate (%)
0.0 ± 0.0	20.4 ± 4.3	34.3 ± 2.5	6484 ± 2300	21.6 ± 9.5 ^a^
474.1 ± 2.6	25.0 ± 1.8	39.7 ± 3.9	7973 ± 1264	24.9 ± 8.0 ^a^
930.2 ± 0.1	25.00 ± 2.8	28.0 ± 2.9	8113 ± 3214	25.6 ± 13.3 ^a^
1396.1 ± 0.1	24.4 ± 2.4	28.9 ± 2.4	6919 ± 1429	44.4 ± 15.3 ^b^

^1^ IC: Initial concentration.

## Data Availability

All data are presented in this article in the form of figures and tables.

## Appendix A

This part gives the code of RF modeling between decay rate and the influence factors to analyze the contributions or importance of input variables. The version of Python 3.10 was used with the integrated development environment of PyCharm. The invoking packages included numpy 1.22.4, pandas 1.3.4, scikit-learn 0.4.2, matplotlib 3.4.3, and scipy 1.7.3.

# -*- coding = utf-8 -*- import numpy as np from sklearn.model_selection import cross_val_predict, KFold from sklearn.metrics import mean_absolute_percentage_error import pandas as pd from sklearn.ensemble import RandomForestRegressor from sklearn.cluster import AgglomerativeClustering import matplotlib.pyplot as plt from scipy.cluster.hierarchy import dendrogram, linkage plt.rcParams[‘font.sans-serif’] = [‘SimHei’] xlsx_name = ‘Aerosol Data.xlsx’ # Read the xlsx file data = pd.read_excel(xlsx_name) # Convert data to DataFrame df = pd.DataFrame(data) # Split feature columns and target column X = df.iloc[:, 1:6] # Feature columns y = df[‘Decay rate k, s-1’] # Target feature column # Initialize the random forest regressor rf = RandomForestRegressor(n_estimators = 100, random_state = 42) # 10-fold cross-validation kf = KFold(n_splits = 10, shuffle = True, random_state = 42) # Make predictions y_pred = cross_val_predict(rf, X, y, cv = kf) print(y_pred) # Read the original data df = pd.read_excel(‘output.xlsx’) # Write the list to the 7th column of the Excel file df[‘Predicted Value’] = y_pred # Save data to Excel file df.to_excel(‘output.xlsx’, index = False) # Calculate the mean absolute percentage error mape = mean_absolute_percentage_error(y, y_pred) # Visualize the comparison of predicted values and true values plt.figure(figsize = (10, 6)) plt.plot(np.arange(len(y)), y, label = ‘True Values’, color = ‘b’) plt.plot(np.arange(len(y)), y_pred, label = ‘Predicted Values’, color = ‘r’) plt.xlabel(‘Sample Number’) plt.ylabel(‘Target Feature Value’) plt.title(‘Comparison of True and Predicted Values’) plt.legend() plt.show() print(f’Mean Absolute Percentage Error: {mape}’) # Feature Importance Analysis model = RandomForestRegressor() model.fit(X, y) feature_importances = model.feature_importances_ # Analyze the influence/contribution of each factor on the target feature_importance = pd.Series(model.feature_importances_, index = X.columns).sort_values(ascending = False) print(‘Influence/Contribution of Each Factor on the Target:’) print(feature_importance) # Hierarchical Clustering Analysis clustering = AgglomerativeClustering(n_clusters = 3, affinity = ‘Euclidean’, linkage = ‘ward’) clusters = clustering.fit_predict(X) # Hierarchical clustering analysis based on the degree of influence of different factors Z = linkage(X.T, ‘ward’) plt.figure(figsize = (10, 5)) dendrogram(Z, labels = X.columns) plt.title(‘Hierarchical Clustering Dendrogram’) plt.xlabel(‘Features’) plt.ylabel(‘Distance’) plt.show()
